# Effect of a Daily Collagen Peptide Supplement on Digestive Symptoms in Healthy Women: 2-Phase Mixed Methods Study

**DOI:** 10.2196/36339

**Published:** 2022-05-31

**Authors:** Mariette Abrahams, Rochez O’Grady, Janne Prawitt

**Affiliations:** 1 Qina Ltd Olhao Portugal; 2 Rousselot BV Gent Belgium

**Keywords:** collagen peptides, collagen hydrolysates, digital study, gut, digestive symptoms, technology, bloating, Peptan, microbiome, health care professionals, mobile phone

## Abstract

**Background:**

The effect of dietary collagen on managing digestive symptoms is currently lacking in the literature.

**Objective:**

To gain a better understanding of this issue, we conducted a 2-phase mixed methods study.

**Methods:**

Phase 1 was a mixed methods design to explore current attitude and practice among consumers and health care practitioners. The findings were used to design an 8-week phase 2 digital study called Gutme! conducted in the United States in healthy female volunteers (BMI>25 kg/m^2^). Our aim was, first, to determine the feasibility of conducting a fully digital mixed methods study; second, the study explored the effect of an 8-week daily supplementation of 20 g dietary collagen peptide (Peptan) on digestive symptoms. Phase 2 was a prospective, open-label, longitudinal, single-arm study. Participation involved 2 weeks of baseline tracking (digestive symptoms, mood, stool, and lifestyle) using an app, followed by 8 weeks of tracking and taking 20 g collagen peptide supplement split into 2 dosages per day. Participants were required to complete a web-based symptom questionnaire at baseline, week 2, and week 8, as well as participate in 2 scheduled video interviews.

**Results:**

Phase 1 revealed that consumer awareness of collagen for digestive health is low (64/204, 31.4%). Among the dietitians prescribing collagen for their patients, the most common dosage was 20 g a day with notable effects after 6 weeks of intake. Within the phase 2 study, of the 40 recruited participants, 14 (35%) completed the full course of supplementation. The findings indicate that 93% (13/14) of those who completed the study experienced a reduction in digestive symptoms, which included bloating.

**Conclusions:**

A mixed methods digital study design is feasible and acceptable for collecting relevant data in a real-life setting. The use of a 20 g daily collagen peptide supplement may reduce bloating and improve mild digestive symptoms in otherwise healthy female adults in the absence of any other dietary or lifestyle interventions.

**Trial Registration:**

ClinicalTrials.gov NCT04245254; https://clinicaltrials.gov/ct2/show/NCT04245254

## Introduction

### Background

Disorders of gut-brain interaction (DGBIs), formerly known as functional gastrointestinal disorders (FGIDs), relate to an array of gastrointestinal (GI) disorders, with irritable bowel syndrome and functional dyspepsia being the most common. Symptoms of DGBIs include bloating, stomach cramps, pain, diarrhea, constipation, flatulence, irregular bowel movements, and acid reflux. Most recently, a Rome Foundation Global Study estimated that >40% of the global population experiences at least one digestive disorder, with similar statistics in the United States alone (39.9%) [1]. Women are disproportionately affected, with an odds ratio of 1.7 compared with men [1]. A possible explanation for this phenomenon is that women are more likely to discuss digestive symptoms with their medical doctor than men [2]. Although uncomfortable digestive symptoms commonly do not warrant a medical visit or hospitalization, they can affect quality of life [3], and the economic impact cannot be ignored, with recent findings demonstrating that the direct and indirect costs, including absenteeism and productivity, in the United States were US $21 billion or the equivalent of US $500 to US $1200 per patient per annum [3].

The etiology for DGBIs is not always very clear. On the basis of the biopsychosocial model for FGIDs from the Rome IV study, potential explanations include an interplay among the following factors: genetics, psychological factors (early life trauma, psychological state, and social support), disturbance in gut-brain axis, and environmental stressors (infection, use of antibiotics, and poor dietary habits such as a low-fiber diet) [4].

Despite its high prevalence, for many decades the management of what was formerly known as FGIDs revolved around treating specific gut symptoms with over-the-counter medications, many without credible scientific data backing their efficacy [5]. The cause of digestive symptoms can be multifactorial; current practice is to use standard approaches based on the diagnosis. Examples of current approaches include cognitive behavioral therapy [6]; a diet low in fermentable oligo-, di-, and monosaccharides and polyols [7]; over-the-counter solutions such as fibers and gels; and lifestyle changes [8]. Dietary supplements and functional food products, including probiotics, vitamins, L-glutamine, and antacids, are additional approaches commonly chosen by consumers to help them manage their symptoms [9]. Most recently, dietary collagen in the form of collagen peptides or collagen hydrolysates have been added to this supplement selection. Collagen peptides are sourced from bovine, porcine, marine, or poultry sources [10]. Although there are positive reports of the use of collagen supplementation in skin hydration as well as joint, bone, and muscle health, especially in athletes to aid exercise recovery, reports of the benefits of the use of collagen in digestive health are currently lacking [11-15].

The intestinal epithelium acts as an active barrier between the external and internal environments, protecting the organism from toxins as well as regulating intestinal homeostasis and nutrient absorption [16]. Tight junctions (TJs) are an integral part of these paracellular barriers, it having become clear in recent years that they are responsible for more functions beyond simple diffusion. Their proteins have been shown to be used by viruses and pathogenic bacteria to gain access across the barrier into the cells; moreover, mutations in the genes encoding these proteins have been linked to an array of inherited diseases [17]. Dysregulation of the intestinal barriers can be multifactorial, with infection and inflammation playing a part [16]. This can lead to increased gut permeability, which has also been discovered in several GI disorders such as Crohn disease and described as a contributing factor in pathogenesis of one of the most common DGBIs, irritable bowel syndrome [18,19]. TJs are affected by inflammation and proinflammatory cytokines, in particular, elevated levels of tumor necrosis factor-alpha, and this has been shown to disrupt the barriers, leading to increased permeability [20]. Research regarding the effect of collagen peptides on gut permeability through different mechanisms of action is limited and restricted to animal models or cells [21-23]. In the study by Chen et al [16], collagen peptides derived from the skin of the Alaska pollock significantly attenuated the tumor necrosis factor-alpha–induced dysfunction of the TJ barrier in Caco-2 cell monolayers. These interesting findings need to be explored further; however, this may be an indication that collagen peptides might be helpful in targeting gastric and bowel-related issues. The goal of this paper was to explore the potential role collagen could play in the management of common digestive symptoms such as bloating.

Despite the best intentions, traditional study designs may not sufficiently detect interindividual differences [24] and are often far removed from real-life settings [25]. New approaches and study designs as well as statistical methods are now recommended to better identify and detect interindividual differences in response to supplements, ingredients, or interventions [26]. These new study approaches leverage digital technologies, such as apps, wearables, trackers, and smartwatches, that have opened up the opportunity to reach wider groups and offer an efficient and convenient way to collect valuable data in the real-world context [27]. With common digestive symptoms in particular, apps can be useful to identify the often hidden relationship among food, mood, lifestyle, and digestive symptoms, which can then be used to personalize dietary and lifestyle advice accordingly [28]. Advanced analytics such as machine learning or artificial intelligence can then identify patterns or trends in the data shared or submitted by users [29].

### Objectives

The main aim of the Gutme! study was to assess the feasibility and acceptability of a mixed methods digital study.

The secondary aims were set for each phase as follows:

- The aim of the first phase of the study was to obtain a better understanding from consumers and frontline health care practitioners on how they use collagen to manage digestive symptoms such as bloating and explore their perceptions on how collagen works.
- The aim of the second phase of the study was to determine the response to a collagen peptide supplementation to reduce digestive symptoms in otherwise healthy consumers.

The purpose of the study was to determine whether a mixed methods study design could be used to collect reliable data in a real-life setting and whether collagen peptides could be potentially used as a solution in the management of mild digestive symptoms in healthy female adults.

Our hypothesis was that an 8-week supplementation with bovine-based collagen peptides would reduce digestive symptoms in healthy female adults.

## Methods

### Study Design

**Figure 1:**
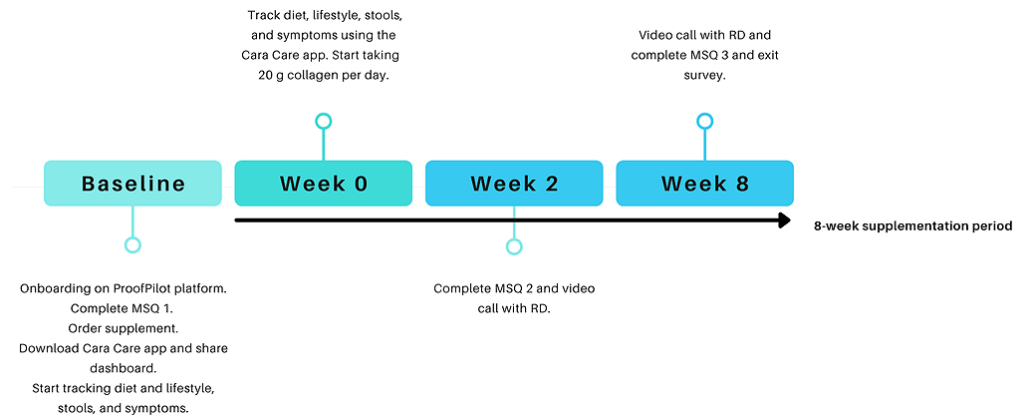
Phase 2 study flowchart. Baseline relates to a 2-week preintervention phase with variable tracking: diet and lifestyle, stools, symptoms, and Medical Symptoms Questionnaire (MSQ) 1; week 0-8 relates to an 8-week-long collagen supplementation with variable tracking; MSQ 2 was administered 2 weeks after supplementation; and MSQ 3 was administered 8 weeks after supplementation. RD: registered dietitian.

The phase 1 consumer study, which was led by a third party based in the United Kingdom (Streetbees), included a web-based survey that used an artificial intelligence approach. Web-based interviews were conducted by a Portugal-based consultancy (Qina) with a small group of functional and integrative nutrition registered dietitians (RDs) based in the United States who used collagen peptides for the management and treatment of digestive complaints. The phase 2 study used a prospective, open-label, single-arm, longitudinal design. The full study design flow is outlined in Figure 1.

### Ethics Approval

The study design was approved by Advarra, an ethics advisory board (2329-ProofPilot), and was registered on ClinicalTrials.gov (NCT04245254). The Gutme! study ran between March 2020 and October 2020 during the COVID-19 pandemic.

### Inclusion and Exclusion Criteria

#### Phase 1

The inclusion criteria were as follows: the consumer group, as determined by Streetbees, included participants actively taking collagen supplements, were based in the United States, had access to the Streetbees app, and were aged 18 to 65 years. Current or past medical history was not part of the screening. The RD group included functional and integrative nutrition RDs based in the United States who were using collagen in their practice.

#### Phase 2

The inclusion criteria were as follows: women aged 35 to 65 years with access to the internet and experiencing 3 out of the following 5 symptoms: bloating, flatulence, acid reflux, stomach pain, and irregular bowel movements; app or smartphone user; English speaking; based in the United States; and with no self-reported medical diagnosis and not on medication (with the exception of oral contraceptives). Age range was determined from the interviews with the RDs in phase 1 who reported that the majority of their clients with GI symptoms were aged 35 to 65 years.

The exclusion criteria were as follows: participants with <3 digestive symptoms, chronic kidney disease, scleroderma, allergy to glutamate and beef, or kidney stones. This was based on feedback from the RDs in the phase 1 study. In addition, we excluded participants who were already taking collagen, had recent antibiotic use, or experienced occurrence of DGBIs (eg, Crohn disease) or ulcerative colitis.

In terms of quality criteria, participants who did not respond to emails after 5 attempts were considered dropouts. Study completion was considered at 9 weeks. In addition, on the ProofPilot platform, no more than 2 users per physical address were allowed to participate.

### Participant Recruitment

With no prior data to base the recruitment numbers on, power calculations were not performed for phase 2; to start the study, the goal was set arbitrarily at n=40. Participants were recruited through social media advertisements on Facebook and Instagram, web-based forums, professional networks, social media influencers, and private gut health groups, as well as the ProofPilot platform. On the basis of the statistics provided by the advertising platforms, recruitment reached >20,000 participants, and the recruitment goal was reached at 8 weeks.

### Study Materials (Phase 2)

Peptan (Rousselot) was the collagen peptide brand used as the intervention. The powdered supplement mixes easily into food and beverages and is widely available on the market. The collagen peptides were supplied by the manufacturer as 10 g stick packs, each batch coded and labeled for the entire study period and delivered directly to each participant’s home through a courier service. Participants were instructed to take 20 g per day (as 2×10 g divided doses), mixed in food or beverages. Participants were aware that they were taking collagen peptides; however, they were not told which brand was being used. Maltodextrin was the only potential candidate as a control supplement; however, because of its deleterious effect on gut health [30], an alternative was to enable participants to track the effect of supplementation through a 2-week baseline assessment of their usual food intake, physical activity, and symptoms.

The Medical Symptoms Questionnaire (MSQ) is a functional medicine tool used to measure self-reported symptoms, which helps to identify potential health issues within various physiological and emotional body systems (Multimedia Appendix 1) [31]. Administering this questionnaire was the preferred method of collecting data by the RDs in their practice to track their clients’ progress over time. The questionnaire consists of 71 questions, with each symptom scored on a 5-point (0 to 4) Likert scale from *Never or almost never have the symptom* to *Frequently have it, effect is severe*. The decision to use the questionnaire was based on the phase 1 study, which found that the tool was used by the majority of RDs in their clinical practice. The MSQ was administered as a web-based survey directly on the ProofPilot app to all eligible and consented participants at baseline as well as at week 2 and week 8 after supplementation. The survey took 10 to 15 minutes to complete.

The Cara Care app is a digital therapeutic app for use by individuals experiencing digestive symptoms. The app is a certified Class I medical device that has been approved as a digital therapeutic for the management and treatment of digestive disorders by Germany’s Federal Institute for Drugs and Medical Devices [32]. The app consists of features for logging and tracking meals, gut symptoms, stool, mood, bowel regularity, menstruation, water consumption, and physical activity. The app has a dashboard feature that can be shared with the research team for viewing submitted data in real time. The dashboard consists of a visual chart that indicates how total scores for bloating and stool symptoms track over time. In this study, we used the free version of the app, which does not provide personalized feedback.

Bloating score on the app refers to a self-reported assessment of fluid retention based on a scale of 1 to 100 and is categorized into the following groups: none (0), mild (25), moderate (50), severe (75), and extreme (100).

Stool score on the app is based on the Bristol Stool Chart [33] and refers to the physical appearance of the feces reflecting intestinal transit time. The stool score used in this study ranges from 0 reflecting no bowel movement on a particular day through 7 different categories of stool appearance from constipation (14) to diarrhea (100): separate hard lumps (14); lumpy and sausage-like (28); sausage shape with cracks in surface (42); perfectly smooth, soft sausage (57); soft blobs with clear-cut edges (71); mushy consistency with ragged edges (85); and liquid consistency with no solid pieces (100). Stool frequency refers to the number of stools each participant reported during a 7-day period.

A recent study has demonstrated that using the Cara Care app leads to a reduction of digestive symptoms and improves quality of life [34].

ProofPilot is a digital research platform that is compliant with both the Health Insurance Portability and Accountability Act and General Data Protection Regulation. The platform has a web interface for researchers to track data in real time. Study participants used the platform as an app downloaded onto their smartphone. Participants received reminders to complete study tasks directly on their smartphone during the entire study period.

Google Meet with enhanced security was used to conduct secure video calls with participants at week 2 and week 8 after supplementation.

### Study Procedure

#### Phase 1

The survey was completed by eligible participants directly on the Streetbees app. The questions consisted of a mixture of Likert scale and free-text questions. Participants were also asked to upload images or videos of the nutritional supplements they were taking. Out of the total cohort taking collagen supplements, those who took collagen to manage their digestive symptoms were identified. For the interviews, eligible RDs were identified and contacted through email and a mutually convenient time was set up to conduct the recorded video interviews, each of which lasted up to 60 minutes. All video interviews were transcribed and checked for quality.

#### Phase 2

During the recruitment phase, interested participants were directed to the Gutme! study landing page on the ProofPilot platform, which provides details of the study. If participants were interested in further information, they were required to set up an account on ProofPilot, at which point data regarding their date of birth, location, and gender were collected, as outlined in Figure 1. After registration, all participants were required to complete the eligibility questionnaire.

Immediately after taking the web-based eligibility questionnaire, participants were told about their suitability for the study. Participants then received an email message to confirm their acceptance and were served with an e-consent form at that point. Only consented participants could continue with the tasks that followed. Participants who were not eligible were informed through an email; they were offered the opportunity to scrutinize the ProofPilot study page to participate in any other available studies that were recruiting.

Consented participants were onboarded using a welcome voice message and downloadable instructions that included *How to mix the collagen* and *How to download the Cara Care app*. The first task for onboarded participants was to complete a baseline questionnaire (MSQ 1), after which participants were asked to download the Cara Care app and order their supplements, which were provided free of charge.

Participants were then given instructions to share their Cara Care app dashboards and track their symptoms to obtain baseline measures on what was considered normal for them over the first 2 weeks. Participants were asked to continue with their habitual food intake and exercise for the duration of the study. During this initial 2-week period, participants would have received their supplement delivery. They were then requested to continue tracking their symptoms daily and take the collagen peptide supplement (20 g total per day) as 10 g twice daily, mixed into either food (such as yogurt or oats) or beverages (water or coffee) for the remaining 8 weeks (corresponding to week 0 to week 8). All participants received daily encouraging and informational messages for the duration of the study as a reminder to track their symptoms and take the collagen based on best practice guidelines [35]. Participants were invited to schedule a video call with an RD at week 2 and week 8, to clarify concerns, give feedback, or ask questions. Participants did not receive any dietary or lifestyle advice either on the web or during the video calls, but they were given guidance on how to mix the supplement and log their data. Weekly email reminders were sent through the ProofPilot app to complete assigned study tasks. All participants completed a final web-based exit survey to provide feedback on their experience.

### Statistical Analysis

Data are expressed as mean and 95% CI or mean and SD. As this is a pilot study and no power calculations were performed ahead of the study, the statistical analysis is exploratory, with no *P* values reported. Instead, percentage change was calculated to report changes between treatment weeks.

### Treatment Effect Size

Treatment effect was calculated using the Hedges *g* formula, which comprises the Cohen *d* formula adjusting for within-group estimates of repeated measures variables from baseline and week 8 with 95% CI. The Cohen *d* formula for treatment effect gives biased estimates of the population size because it is based on sample averages (also referred to as uncorrected effect size) [36]. Hedges *g* corrects for the size of the treatment groups and is therefore better suited to repeated measures as used in the study. On the basis of the Cohen assumption, the effect size can be interpreted as follows: 0.2, *small* effect size; 0.5, *medium* effect size; and 0.8, *large* effect size [36]. The full formulas are illustrated below:



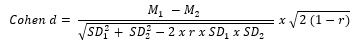





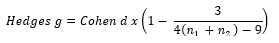



where M_1_: mean of group 1; M_2_: mean of group 2; SD_1_: SD of group 1; SD_2_: SD of group 2; n_1_ and n_2_: number of participants in each treatment; and r: correlation between group 1 and group 2.

## Results

### Results of Phase 1 Study

#### Consumer Survey

Of the 204 consumers who participated in the survey, 35 (17.2%) were collagen users. Collagen users tended to be educated to a higher level and were in the higher income bracket (Figures S1 and S2, respectively, in Multimedia Appendix 1) compared with the rest of the Streetbees app users. The results demonstrate that awareness of collagen for digestive health is low, with only 31.4% (64/204) answering *Yes* and 63.7% (130/204) answering *No* to the question *Before today, had you ever heard of collagen supplements being used to help with digestive health?* The most common reasons for using collagen in digestive health included better digestion, better stomach, and less bloating (Table 1).

Responding to the question *How often do you use your collagen supplement?*, 63% (22/35) indicated that they used it 1 to 2 times per day mostly in powder format and mixed into water (Table 2), which correlated well with the recommendations from practitioners (see *Practitioner Interviews* section).

**Table 1:** Participants’ responses regarding reasons for using collagen (N=35).

What do you think this product does for you?	Participants, n (%)
I feel better overall	9 (26)
Helps with digestion	8 (23)
Keeps me regular	6 (17)
Helps with bloating	2 (6)
Helps with bowel movements	2 (6)
Maintains gut health	2 (6)
Reduces or relieves pain	2 (6)
Helps me to sleep and relax	1 (3)
Helps with constipation	1 (3)
Helps with weight control	1 (3)
Reduces heartburn or acid reflux	1 (3)

**Table 2:** Participant responses to the question *How often do you take collagen?* (N=35).

Responses	Values, n (%)
Multiple times a day	1 (3)
1 to 2 times a day	22 (63)
A few times a week	9 (26)
Less often	1 (3)
I only use it when I am experiencing digestive issues	2 (6)

#### Practitioner Interviews

Interviews with US-based practitioners (n=15) revealed positive outcomes when starting patients and clients on collagen peptides, which included reduction in bloating, stomach cramps, flatulence, and acid reflux, as well as improvement in irregular bowel movements when used daily for a period of at least 6 to 8 weeks. The most common dosage recommended by practitioners was 20 g of collagen peptides per day in powder format, irrespective of the brand. It is important to note that collagen peptides were used as part of a comprehensive supplementary regimen that could include micronutrients, digestive enzymes, glutamine, colostrum, prebiotics, and probiotics. It would therefore be difficult to conclude that an improvement in digestive symptoms was due to collagen alone. A summary of the phase 1 study with practitioners is presented in Table 3.

The first phase provided a clear picture of how and why collagen was being used to manage digestive symptoms, which provided the basis for designing the phase 2 study.

**Table 3:** Main findings of the phase 1 study interviews with registered dietitians (N=15).

Theme	Findings	Quotes
Indication for starting a collagen supplementation	Most started their clients on collagen supplementation based on careful assessment of digestive problems	“So it’s everything from GI^a^ symptoms, from constipation, diarrhea, bloating, [...] reflux [...]”
Recommended dosage	20 g per day as separated doses	“I definitely want people to have at least 12 grams a day. You probably have benefits right up through 25 grams a day”
Duration	6 to 8 weeks	“I would [...] say six weeks and then check in and see your response at that time”
Target group	Women aged 35 to 65 years	“[...] I would say 40s to 60s, but that’s certainly not to say that I haven’t started seeing more and more people in their early 30s”
Perceived benefits	Reduces bloating; heals gut lining; improves digestion	“It depends on the person, most people love continuing on them because not only are you getting the gut healing benefits from collagen [...]”
Monitoring progress	MSQ,^b^ diary, repeat stool test	“We’re using one from the Institute of Functional Medicine and that one’s called the MSQ medical symptom questionnaire”

^a^GI: gastrointestinal.

^b^MSQ: Medical Symptoms Questionnaire.

### Results of Phase 2 Study Gutme!

#### Participant Recruitment

**Figure 2:**
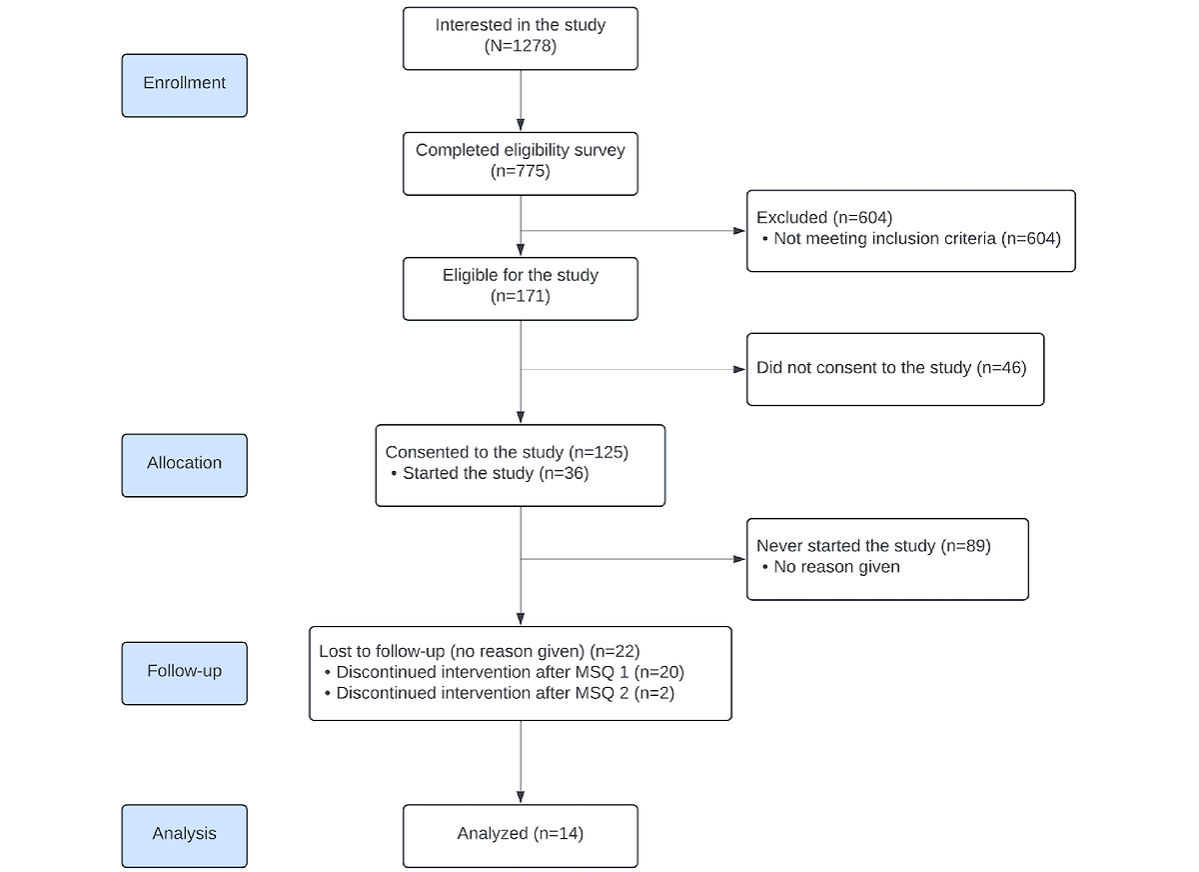
CONSORT (Consolidated Standards of Reporting Trials) diagram with study recruitment figures. MSQ: Medical Symptoms Questionnaire.

Of the 775 individuals who completed the eligibility survey, 604 (77.9%) were not eligible and 171 (22.1%) were eligible. Of these 171 individuals, 46 (26.9%) did not consent. Although the target for recruitment was set at n=40, the platform was left open to allow additional eligible individuals to participate to account for attrition. In total, 125 individuals consented; however, 89 (71.2%) did not start trial participation, leaving 36 (28.8%) who started the study. Of these 36 participants, 22 (61%) were lost to follow-up (n=20, 91%, after MSQ 1 and n=2, 9%, at 2 weeks after supplementation [MSQ 2]). Reasons for not participating were not investigated. Figure 2 shows the CONSORT (Consolidated Standards of Reporting Trials) diagram with study recruitment figures.

#### Study Retention

At baseline and after consenting, the participants (n=36) entered the Gutme! study. By week 4, of the 36 participants, 20 (56%) dropped out, leaving 16 (44%) participants. By week 8, of the 16 participants, 2 (13%) dropped out, leaving 14 (87%) participants after supplementation. Reasons for the high dropout rate include the disruption caused as a result of the COVID-19 pandemic; of the 36 participants, 1 (3%) was diagnosed with a medical condition; a few participants (n=3, 8%) withdrew because of unexpected medical and family emergencies related to COVID-19; however, many participants (n=18, 50%) either failed to respond to emails or withdrew for unknown reasons.

#### Participant Characteristics and Supplement Use

All participants who completed the study were women with an average age of 46 (SD 6.2; range 37-58) years and average BMI of 28 (SD 7.8; range 21-48) kg/m^2^. None of the participants had any medical conditions or were on medication. Ethnicity breakdown of the 14 participants was as follows: White: 9 (64%); African American: 3 (21%); Hispanic or Latino: 1 (7%); and other: 1 (7%). None of these participants had been taking collagen supplements; 14% (2/14) took vitamin and mineral supplements, 28% (4/14) took probiotics, and 21% (3/14) took digestive enzymes.

#### Baseline Symptom Characteristics

Participants regularly experienced all digestive symptoms as outlined in Table 4.

**Table 4:** Symptom occurrence at baseline (N=14).

Symptom	Participants, n (%)
Bloating	14 (100)
Acid reflux	9 (64)
Flatulence	14 (100)
Irregular bowel habits	14 (100)
Stomach cramps	10 (71)

#### Change in MSQ Score

**Figure 3:**
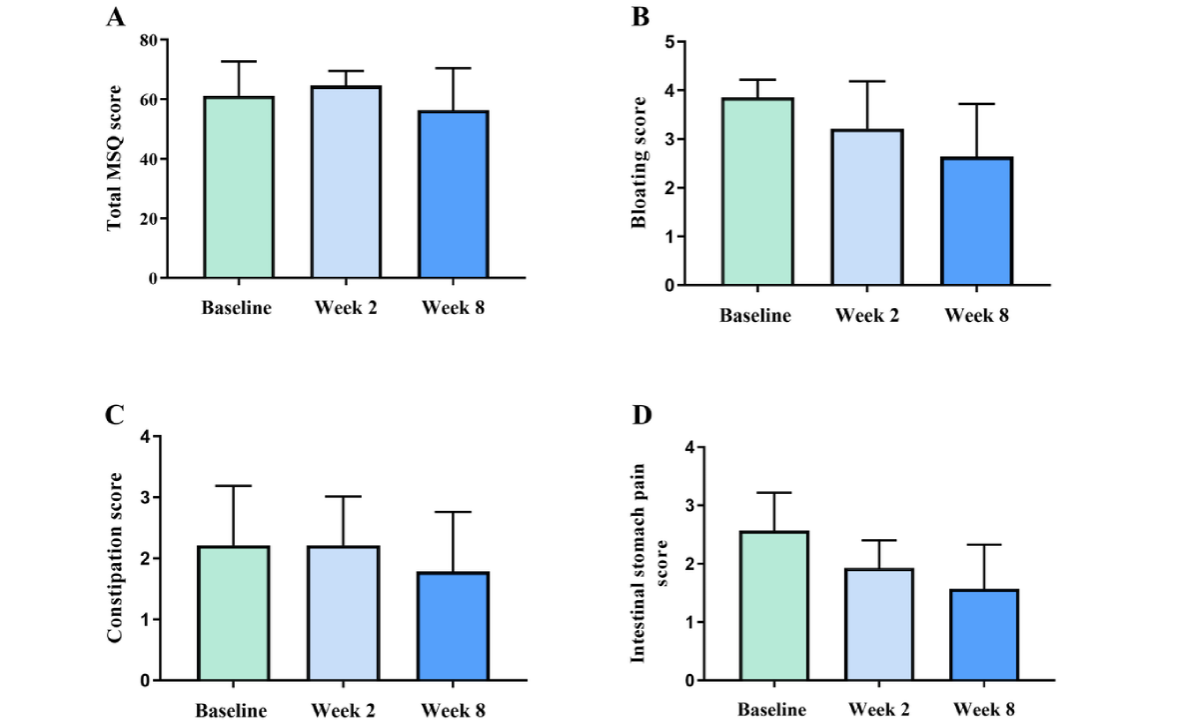
Absolute change in Medical Symptoms Questionnaire (MSQ) scores, n=14: (A) total MSQ score over the study period. Questionnaire administered: MSQ 1 at baseline, MSQ 2 at week 2, and MSQ 3 at week 8 after supplementation; (B) average bloating score; (C) average constipation score; and (D) average intestinal stomach pain score. Average scores in figures B, C, and D were calculated as subscore of digestive health category within the MSQ 5-point scale of symptom occurrence and its effect: 0=never or almost never; 1=occasional but not severe; 2=occasional, severe; 3=frequent, not severe; and 4=frequent, severe. Data are expressed as mean (SD).

Symptom occurrence at baseline is shown in Table 4. The change in MSQ scores for total MSQ and specific symptoms is shown in Table 5. On average, participants reduced their MSQ scores by 4.8 (SD 15.2) points, which indicates a slight improvement in their overall symptoms. On subscore analysis (Figure 3), bloating scores reduced from an average of 3.9 to 2.6 (31%) after an 8-week supplementation, with a trend for reduction in bloating after 2 weeks of supplementation; constipation dropped from 2.2 to 1.8 (19%), indicating an overall improvement. Intestinal stomach reflux reduced from 2.6 to 1.6 (39%), with a substantial reduction after 2 weeks; acid reflux reduced from 2.1 to 1.6 (21%) after 8 weeks.

**Table 5:** Absolute and percentage change in Medical Symptoms Questionnaire (MSQ) scores.

	Baseline score, mean (SD; 95% CI)	Week 2 score, mean (SD; 95% CI)	Week 8 score, mean (SD; 95% CI)	Change, baseline to week 8 (%)
Total MSQ score	61.2 (11.1; 54.4-67.9)	64.6 (4.7; 61.8-67.5)	56.4 (13.5; 48.3-64.5)	–8
Bloating	3.9 (0.4; 3.7-4.1)	3.2 (1.0; 2.7-3.8)	2.6 (1.1; 2.0-3.3)	–31
Constipation	2.2 (1.0; 1.7-2.8)	2.2 (0.8; 1.8-2.7)	1.8 (1.0; 1.2-2.4)	–19
Intestinal stomach pain	2.6 (0.7; 2.2-3.0)	1.9 (0.5; 1.7-2.2)	1.6 (0.8; 1.1-2.0)	–39
Acid reflux	2.1 (1.0; 1.5-2.7)	2 (1.0; 1.5-2.6)	1.6 (1.2; 1.0-2.3)	–21

#### Effect Size

Results of the effect size calculations are shown in Table 6. Total MSQ score, constipation score, and acid reflux score fall within a *small* effect size (<0.5), whereas changes in bloating and intestinal stomach pain scores fall within a *large* effect size (>0.8) as per the Cohen assumptions.

**Table 6:** Effect size.

	Hedges *g*^a^	Lower limit	Upper limit	*r^b^*
Total MSQ^c^	0.36	–0.3	1.0	0.25
Bloating	1.1	0.7	2.3	0.64
Constipation	0.41	–0.3	1.1	0.21
Intestinal stomach pain	1.3	0.6	2.2	0.38
Acid reflux	0.37	–0.1	0.9	0.63

^a^Hedges *g* is calculated from Cohen *d* accounting for repeated measures. Effect size is based on the Cohen assumptions: *g*=0.2 would be considered a *small* effect size, 0.5 represents a *medium* effect size, and 0.8 a *large* effect size.

^b^*r*: Pearson correlation.

^c^MSQ: Medical Symptoms Questionnaire.

#### Cara Care App Data

The Cara Care app logging rate for the 10-week study period was overall very poor. This has resulted in inconsistent data and inconclusive results for a cohort analysis. Reasons for not logging regularly included a difficult interface on the app, it was time consuming to log dietary intake, and participants forgot to log in on a daily basis despite receiving reminders. When participants did provide very occasional food diary entries, they were reviewed by an RD and compared against the national healthy eating guidelines. The diaries indicated that foods selected and consumed suggested a standard American diet high in processed food, low in fiber, and high in fat [37].

Of note, individual variability in symptom scores is demonstrated for a small number of participants in Figures S3 and S4 in Multimedia Appendix 1.

#### Self-reported Data From Interviews and Exit Survey

The exit survey indicated that participants were overall happy with participating in an entirely digital study. When asked where participants had heard about the study, 71% (10/14) indicated friends or family, 14% (2/14) indicated Twitter, 7% (1/14) indicated ProofPilot, and 7% (1/14) had heard about it from a health care professional. Motivation for joining the study included the following: keeping participants busy during COVID-19, 36% (5/14); trying a new supplement, 50% (7/14); and convenient to participate, 14% (2/14). Finally, 64% (9/14) of the participants indicated that they would have been happy to participate if the study was longer: “I found it very eye opening. It made me realize what foods were my trigger and that I needed to change my eating behavior.”

The video interviews and exit surveys provided detailed insight and data into the effect of taking the collagen peptide supplement over the study period. Participants reported a difference in their gut symptoms within approximately 2 days of taking the collagen supplement. On the basis of the interviews and the exit survey data, 93% (13/14) of the participants reported a reduction in bloating and 93% (13/14) experienced an improvement in bowel habits with the most frequently reported symptom being relief from constipation:

The product, it did help with my bloating quite a bit, but even more than that, it practically resolved my chronic constipation. Prior to the study I’d have problems with constipation on almost a daily basis, since beginning the product I’ve had issues maybe once or twice. I no longer have that heavy feeling, as I had in the past.

It’s like day and night with my gut. Bloating is down and I now go to the toilet regularly.

Great reduction in bloating. Consistency of stools now normal, first time ever. No more diarrhea. Felt a difference almost from day 2.

## Discussion

### Principal Findings

Collagen peptide supplements have become increasingly popular over the last decade and are estimated to grow into a US $7.5 billion market by 2027 [28]. Although the majority of collagen users are within the hair and beauty market [28], the use of collagen has found its way into consumer health products such as for sport recovery [15]. Technology-assisted personalized nutrition solutions such as web-based diaries and symptom trackers have grown in popularity and have enabled new ways of delivering care, providing information as well as gathering data [37]. It is this personalized nutrition approach that gives practitioners and consumers alike the opportunity to experiment with new supplements and ingredients (or a combination thereof) to track the effects on their own bodies through a range of digital tools and at-home tests [38]. In fact, the COVID-19 epidemic has spurred the update of digital health tools and supplements owing to an increased awareness and interest in health and nutrition [39].

The effect size of the intervention on digestive symptoms has shown that collagen supplementation resulted in a large effect on symptoms of bloating and intestinal stomach pain, which is consistent with feedback received from participants. Bloating is 1 of the 2 most commonly reported digestive symptoms, with distension being the other [40]. Numerous reasons have been provided, from food intolerance to delayed intestinal transit or infection; yet, the cause is individual and most often not related to the amount of gas in the bowels [40]. The Gutme! study findings indicate that collagen may be useful to reduce bloating in women who regularly experience the symptom. It is important to note that all the participants were in the overweight range, which has been associated with a lower microbiome diversity [41] and therefore potentially poses an increased risk of bloating. Constipation is one of the most common digestive symptoms with a global prevalence of 14% [42]. Common causes include consuming a diet low in fiber, medication (such as iron supplements), a lack of physical activity, and dysbiosis of the gut microbiota [43]. Bowel frequency is often poorly characterized and highly personal [44]. Our qualitative findings indicate that of the 16 participants, 15 (94%) increased their bowel frequency and 3 (19%) reported that their bowel frequency increased from historically once per week or less to once per day since starting the collagen supplement. We would have therefore expected to see a bigger drop in the MSQ score, based on the detailed information and dramatic changes that participants reported during the video calls in terms of their bowel habits, but this was not the case. This could be because the questionnaire took time to complete [45], meaning the participants were tired and not careful in scoring or did not remember how they felt at the start. In addition, the symptoms of a participant who regularly experienced floating stools, an indication of possible malabsorption, resolved after taking the collagen supplement. The reasons for this change in bowel habit are not clear and will require further clinical investigation. Potential explanations for the change in bowel frequency could be a shift in the microbiome composition because of the increased protein load [46] or simply because of an increase in water consumption, with the lack thereof being a common reason for constipation [3]. Although we can speculate that collagen peptides act on the microbiome, the mechanism is not clear. It is unlikely that this change came about as a result of gut healing (or resolution of leaky gut), as suspected in our interviews with practitioners, because the effect would take weeks and not days [47]. Furthermore, it is important to note that we did not provide any dietary or lifestyle guidance to participants. Dietary intake data from the Cara Care app suggested that all participants were following a standard American diet high in processed foods and low in fiber [37]. Participants also had low levels of physical activity (data not shown), which could contribute to their gut symptoms. Whether the supplementation would still have the same effect if participants had an overall better baseline diet and gut microbiome requires further investigation.

### Key Learnings From the Study

Digital studies offer the advantage of including volunteers in real-life settings when they are able to follow their normal routines, including diet and exercise. Especially during the pandemic, the number of digital studies conducted have increased and can offer significant advantages, in particular with remote site monitoring and recruitment [48]. Social media can be a good way to attract and recruit study participants by means of word of mouth through family and friends for common issues that are often discussed freely on the web. Cross-referencing the data of the Cara Care app, the MSQ questionnaire, and video interviews allowed us to view the data both as a cohort and individually. Furthermore, participants expressed an appreciation of being viewed as equals during the study where they also received information in exchange (an explanation of their Cara Care app dashboard) and were given the opportunity to contribute to the development of a needed solution for women, making digital studies an attractive and convenient option.

### Limitations

This study lacked a control group; hence, including a control group, which could comprise a group of participants receiving standard practice, should be considered for future studies. As it was a pilot study with only 1 arm to assess feasibility, the study was neither randomized nor blinded. These study designs could contribute greatly to obtaining more reliable data on the effect of supplementation and will be taken into account for future studies. During the recruitment process, the study suffered high dropout rates, especially after consent. As previously discussed, the pandemic played a role in the willingness of participants to commit to additional responsibilities; however, this should be considered in future studies. Moreover, the sample size was small and therefore the findings should be interpreted with caution. The study period was long, which could have affected participation and completion rates; future studies could be shorter in duration, especially considering that participants reported much perceived improvement within days of taking the supplement. Selecting participants from different locations with a variety of dietary patterns could provide better insight into the true effect of collagen supplementation for the same digestive symptoms. Our sample only included women, and therefore future studies should also include men. Menstrual cycles are known to affect fluid retention, which has an impact on body composition, with levels peaking on the first day of menstruation [49,50]. This physiological process may have an impact on the effect of supplementation on the gut in studies where bloating is one of the outcomes, such as the study reported here. Another limitation is that the study was limited to participants who were digitally literate and had access to the internet, thereby excluding individuals who were not digitally literate and did not have access to the internet [51].

The completion rate for this study was 35% (14/40), which was mostly as a result of unforeseen circumstances during the COVID-19 pandemic; yet, these could be addressed with better participant screening, monitoring, and engagement or by adopting a hybrid approach. Other digital studies have reported a completion rate between 50% and 80% [52,53]. It was cumbersome for participants to use 2 separate apps (ProofPilot and Cara Care), which led to inconsistent tracking with regard to diet and physical activity. Selecting interoperable solutions that already have popular apps integrated should be a key consideration for future studies. Finally, participants were told that the interviews would be conducted by an RD, and this could have introduced bias in terms of how big an effect collagen supplementation had [54]; however, the results in terms of effect size suggest otherwise.

Future studies should consider including microbiome, genetic, and metabolic data. They should also consider diet quality and lifestyle factors using convenient and noninvasive tracking. Research-grade image-recognition dietary assessment tools could improve the participant experience by reducing time to log meals and provide insight into the quality of their diet. In addition, increasing the ethnic diversity of participants should be a core focus in future research, considering that digestive symptoms are so common and span across ethnicities.

### Conclusions

A mixed methods digital study design is a feasible and acceptable method to explore the effect of a supplement in a real-life setting. In this small 8-week digital study, the consumption of 20 g collagen peptide supplement (Peptan) may have resulted in a reduction in bloating and an improvement in bowel frequency in the absence of any other dietary or lifestyle intervention or advice. These findings warrant confirmation in a larger, well-controlled study with or without dietary guidance.

## Appendix

Multimedia Appendix 1 Medical Symptoms Questionnaire; Figure S1: Education level of phase 1 survey participants who take collagen supplements; Figure S2: Average salary of phase 1 survey participants who take collagen supplements; Figure S3: Bloating score; Figure S4: Stool frequency.

## Abbreviations

CONSORT: Consolidated Standards of Reporting Trials
DGBI: disorder of gut-brain interaction
FGID: functional gastrointestinal disorder
GI: gastrointestinal
MSQ: Medical Symptoms Questionnaire
RD: registered dietitian
TJ: tight junction
