# Supplementary material for: Effect of a Daily Collagen Peptide Supplement on Digestive Symptoms in Healthy Women: 2-Phase Mixed Methods Study
Source: JMIR Form Res. 2022 May 31;6(5):e36339. doi: 10.2196/36339 (PMC9198822; doi:10.2196/36339)
Supplement: Multimedia Appendix 1 [file formative_v6i5e36339_app1.docx]

# **Supplementary Materials**

Supplementary material 1. Medical Symptom Questionnaire (MSQ)

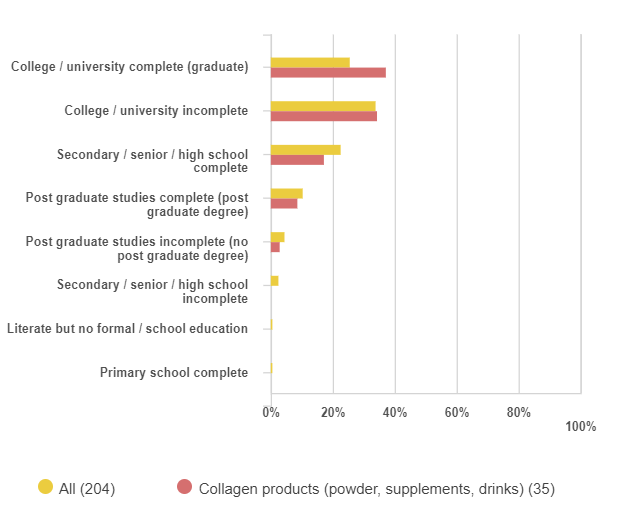


Figure S1. Education level of participants in Phase I survey who take collagen supplements. ‘All’ refers to participants taking collagen for variety of conditions, N= 204; ‘Collagen products’ refers to participants taking collagen supplements for digestive health, N=35.


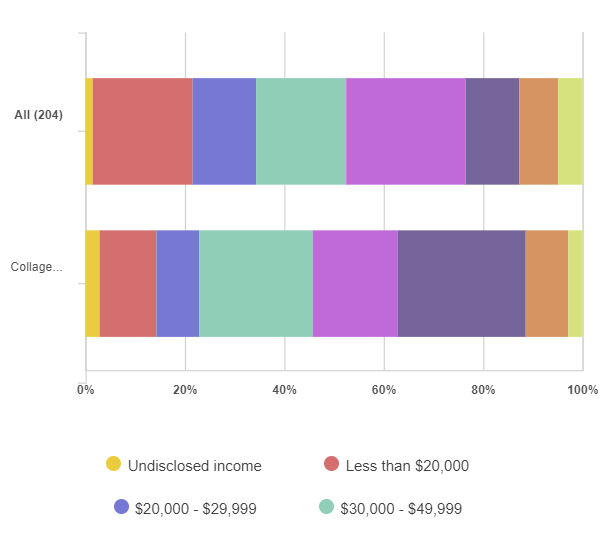


Figure S2. Average salary of participants in Phase I survey who take collagen supplements. ‘All’ refers to participants taking collagen for variety of conditions, N= 204; ‘Collagen products’ refers to participants taking collagen supplements for digestive health, N=35

*
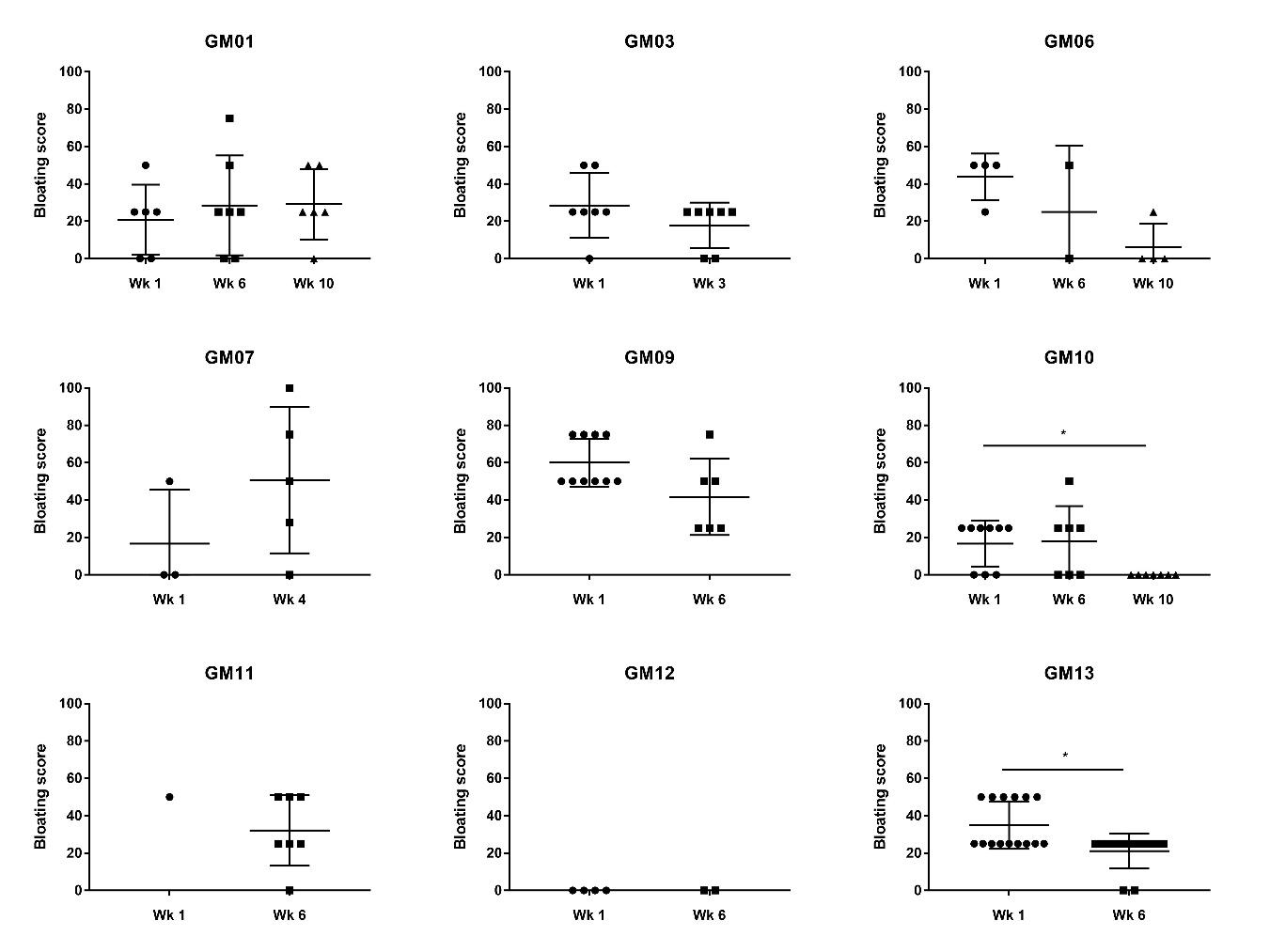
*

Figure S3. Bloating score. Each datapoint refers to a single entry in Cara care app accordingly to a scoring classification: none (0), mild (25), moderate (50), severe (75), extreme (100). GM code refers to a participant number. N=9

*
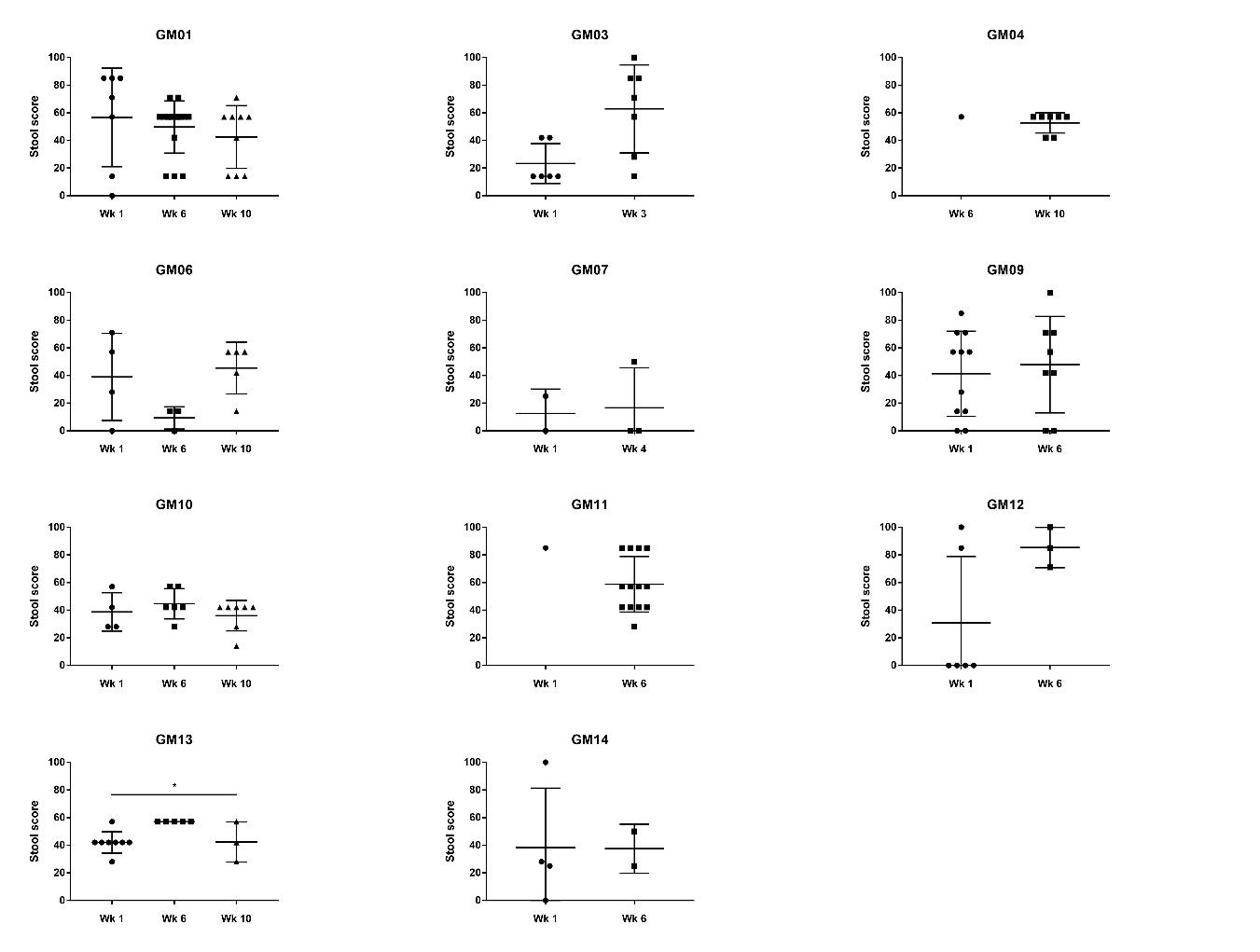
*

Figure S4. Stool frequency. Each datapoint refers to a single entry of bowel movement in the app who entered daily for the duration of the week. N=3
